# To: Improving the outcomes of sepsis in Brazil: strategies and initiatives

**DOI:** 10.62675/2965-2774.20260226

**Published:** 2026-01-14

**Authors:** Vanessa Soares Lanziotti, Saraswati Kache

**Affiliations:** 1 Universidade Federal do Rio de Janeiro Instituto de Puericultura e Pediatria Martagão Gesteira Clinical Research and Health Technological Innovation Unit Rio de Janeiro RJ Brazil Clinical Research and Health Technological Innovation Unit, Instituto de Puericultura e Pediatria Martagão Gesteira, Universidade Federal do Rio de Janeiro - Rio de Janeiro (RJ), Brazil.; 2 Lucile Packard Children's Hospital Department of Pediatrics Division of Critical Care Medicine Stanford California United States Division of Critical Care Medicine, Department of Pediatrics, Lucile Packard Children's Hospital, Stanford Medicine – Stanford, California, United States.

**To the editor**

We read with great interest the article "Improving the outcomes of sepsis in Brazil: strategies and initiatives" by Souza et al.,^(1)^ which presents the significant impact of *Instituto Latino Americano de Sepse* (ILAS). For 20 years, ILAS has successfully implemented strategies for both clinical improvements and collaborative research studies in adult and pediatric sepsis in Brazil and Latin America.

This paper applauds ILAS's efforts and uses them as a launching pad to summarize the efforts that can be implemented in low- and middle-income countries (LMICs) despite limited resources to improve outcomes and research efforts.

In children, sepsis is the primary cause of mortality and pediatric intensive care unit (ICU) admissions worldwide. The data collected while creating the Phoenix Criteria for Pediatric Sepsis and Septic Shock demonstrated a mortality rate that is three times higher in LMICs as compared to high-income settings.^(2)^ The SPREAD PED a 1-day point prevalence study in Brazilian pediatric ICUs conducted by ILAS estimated more than 40 thousand cases of pediatric sepsis and 8,300 deaths per year, confirming the high burden of pediatric sepsis in Brazil.^(3)^ Incomplete vaccinations and nosocomial infections were associated with higher mortality. Although they are amenable to simple public health interventions, such as washing hands and vaccination campaigns, numerous challenges exist, i.e., human factors, economics, and organizational factors.

Multiple other global efforts have focused on pediatric sepsis and are listed below. The Pediatric Acute Lung Injury and Sepsis Investigators (PALISI)^(4)^ network, founded in 2002, has developed multiple North-South partnerships with many efforts focusing on improving outcomes of sepsis patients. The Children's Hospital Association Improving Pediatric Sepsis Outcomes (IPSO) collaborative in the United States was created in 2017 and aims to improve sepsis outcomes through collaborative learning and quality improvement interventions.^(5)^ The Pediatric Sepsis Data CoLaboratory (Sepsis CoLab),^(6)^ in Canada, together with the World Federation of Pediatric Intensive and Critical Care Societies (WFPICCS), promotes international collaboration for clinical data sharing, along with development and validation of new tools to improve pediatric sepsis care. Founded in 2024, the Global Sepsis Alliance^(7)^ launched, in September 2024, the 2030 Global Agenda for Sepsis to improve sepsis outcomes and has engagement from the five continents. The Pediatric Surviving Sepsis Campaign, published for the first time in 2005 and in its last version in 2020,^(8)^ promotes the development of "evidence-based recommendations for clinicians caring for children with septic shock and other sepsis-associated organ dysfunction". It involves the work of nearly 50 global sepsis experts, including two Brazilian representatives from ILAS.

ILAS has established partnerships with the Global Sepsis Alliance and the *Sociedad Latinoamericana de Cuidados Intensivos Pediátricos* (SLACIP- a member society of WFPICCS) and has worked to develop multicenter studies with other institutions and Latin American research networks. These South-South initiatives are crucial for having more accurate sepsis data from the Southern Hemisphere and empowering Global South Research. These initiatives reflect the importance of improving pediatric sepsis outcomes worldwide and illustrate that collaborative efforts, both locally and globally, are crucial to reducing pediatric sepsis burden. ILAS is a model of such efforts for other LMICs.
